# Vertebral Bomb Radiocarbon Suggests Extreme Longevity in White Sharks

**DOI:** 10.1371/journal.pone.0084006

**Published:** 2014-01-08

**Authors:** Li Ling Hamady, Lisa J. Natanson, Gregory B. Skomal, Simon R. Thorrold

**Affiliations:** 1 Biology Department, Woods Hole Oceanographic Institution, Woods Hole, Massachusetts, United States of America; 2 Massachusetts Institute of Technology/Woods Hole Oceanographic Institution Joint Program in Oceanography/Applied Ocean Science and Engineering, Cambridge, Massachusetts, United States of America; 3 Apex Predators Program, National Marine Fisheries Service, National Oceanic and Atmospheric Administration, Narragansett, Rhode Island, United States of America; 4 Massachusetts Division of Marine Fisheries, New Bedford, Massachusetts, United States of America; The Australian National University, Australia

## Abstract

Conservation and management efforts for white sharks (*Carcharodon carcharias*) remain hampered by a lack of basic demographic information including age and growth rates. Sharks are typically aged by counting growth bands sequentially deposited in their vertebrae, but the assumption of annual deposition of these band pairs requires testing. We compared radiocarbon (Δ^14^C) values in vertebrae from four female and four male white sharks from the northwestern Atlantic Ocean (NWA) with reference chronologies documenting the marine uptake of ^14^C produced by atmospheric testing of thermonuclear devices to generate the first radiocarbon age estimates for adult white sharks. Age estimates were up to 40 years old for the largest female (fork length [FL]: 526 cm) and 73 years old for the largest male (FL: 493 cm). Our results dramatically extend the maximum age and longevity of white sharks compared to earlier studies, hint at possible sexual dimorphism in growth rates, and raise concerns that white shark populations are considerably more sensitive to human-induced mortality than previously thought.

## Introduction

White sharks (*Carcharodon carcharias*) are wide ranging apex predators in coastal and offshore waters throughout the world’s oceans. They are considered “vulnerable” worldwide by the International Union for the Conservation of Nature [1] Red List of Threatened Species and are protected via international trade agreements including the Convention on International Trade in Endangered Species [2] and the Convention on Migratory Species [3]. Despite this attention, remarkably little demographic information is available for white shark populations. Age estimation is particularly important to the development of conservation and sustainable management strategies as most demographic variables required for adequate population assessments, such as longevity, growth rate, and age at sexual maturity, include an age component. The primary method of age estimation in fishes relies on counting growth increments in mineralized tissues, including otoliths, vertebrae, and fin rays [4], [5]. However, accurate interpretation of growth increments in these tissues is often difficult and, in the case of a species subjected to fisheries exploitation, misinterpretation may inadvertently lead to mismanagement [6], [7]. It is, therefore, necessary to validate age and growth estimates that are based on increment counts.

The use of bomb radiocarbon to test the periodicity of increment formation and age is now well established and its use on sharks has increased considerably in recent years [4], [5]. The approach takes advantage of the pulse of radiocarbon above natural levels that was produced as a result of atmospheric testing of thermonuclear devices during the 1950 s and ’60 s. This increase in atmospheric radiocarbon, measured as Δ^14^C [8], mixed relatively quickly into the ocean and became incorporated in the tissues of marine organisms through uptake of dissolved inorganic carbon and subsequent dietary transmission through ocean foodwebs. The rapid rise in radiocarbon in the ocean can be used as a time stamp to determine the age of an organism that deposited layers in accretionary structures during this specific time period, and is generally considered the most diagnostic portion for Δ^14^C-based age determination ([4] though see [9]). Estimated ages based on band pair counts can then be independently confirmed by comparing Δ^14^C values from specific increments in the structure to a Δ^14^C reference chronology of known age material, typically from the same or nearby geographic area. First applied to bony fishes by comparing otolith Δ^14^C to a coral reference chronology [10], its use has since been extended to elasmobranch vertebrae [11]–[17].

Several studies have used vertebral band pairs to describe the age and growth of white sharks. Assuming annual deposition of growth bands, the oldest individuals identified to date from the northeastern Pacific [18], [19], western Indian [20], and northwestern Pacific [21] oceans, were 18 (4.61 m total length, TL), 13 (3.73 m pre-caudal length, PCL), and 12 years (4.42 m TL), respectively. Two other papers described counts of 22 and 23 band pairs from the vertebrae of two large females, both over 5 m in total length, from the southwestern Pacific Ocean [22] and western Indian Ocean [23], respectively. None of the studies were, however, able to document annual periodicity of the band pairs used to assign age. Two of the studies [19], [20] attempted to confirm annual periodicity of growth bands in white shark vertebrae, but results were inconclusive.

Our goal in the present study was to determine periodicity of band pair deposition in the vertebrae of white sharks from the northwest Atlantic Ocean (NWA) using the bomb radiocarbon signal. Once validated, band pair counts provide a method for providing minimum estimates of longevity in white shark populations.

## Methods

Vertebrae were sampled from four female and four male white sharks caught in the NWA from 1967 to 2010 and archived at the National Marine Fisheries Service in Narragansett, RI. Vertebrae were loaned with permission to sample. With the exception of one individual (WS81), all vertebrae were taken from the abdominal section of the vertebral column. Abdominal vertebrae were unavailable from WS81 necessitating the use of a tail vertebra. The vertebrae were sectioned using a Ray Tech Gem Saw to approximately 0.6 mm in thickness. Larger vertebrae were sectioned through the corpus calcareum with a diamond blade using a Diamond Pacific Model TC-6 trim saw. Each section was digitally photographed with an MTI CCD 72 video camera attached to a SZX9 Olympus stereomicroscope using reflected light. Reference to trade names does not imply endorsement by NMFS. Two experienced readers (LJN and GBS) independently counted the growth bands. An opaque band through the intermedialia that continued to the corpus calcareum as a translucent band constituted a growth band. Definition of a band pair was similar to those used in earlier studies that confirmed the annual periodicity of band pairs in the porbeagle, *Lamna nasus,* and the shortfin mako, *Isurus oxyrinchus* [11], [24], which are closely related phylogenetically to the white shark.

The white shark vertebrae were characterized by narrow banding patterns that made it problematic to extract enough material from individual band pairs for Δ^14^C analysis. Therefore, sections were measured down the middle of the intermedialia from the central focus to the outer margin. Samples were cut along measured increments using a razor blade (n = 3 to 23 per vertebra) and were aligned with their respective band pairs using annotated photographs of each section. Band pair deposition was initially assumed to be annual in periodicity and ages were assigned to sample sections based on back calculation from collection date. For WS105, the year of collection (1986) sample was thinly shaved from the outer vertebral surface, representing the material most recently deposited prior to the individual’s death.

Radiocarbon analyses (n = 82) were conducted on collagen in the white shark vertebrae. Carbon isotope values in collagen reflect those of protein whereas the calcified inorganic component of vertebrae (hydroxyapatite) is composed of dietary carbon and dissolved inorganic carbon (DIC) [25], [26]. Dietary and DIC pools have distinctive isotope values in ocean environments [27], [28], which can cause problems when conducting bulk isotope analyses of vertebral material. Varying degrees of mineralization along a vertebra may lead to unequal carbon contributions from organic and inorganic pools to different material sampled longitudinally from a vertebra. Finally, Δ^14^C values in de-mineralized samples from white shark vertebrae have been shown to be lower than paired bulk samples [19], presumably due to the presence of carbon sourced from DIC in the bulk samples. Taken together, these observations provide a strong argument for performing collagen extraction before Δ^14^C analysis of vertebral samples.

Collagen extraction from vertebral samples was conducted following Tuross et al. [29]. Each sample consisted initially of approximately 0.5 g of tissue. Treatment was a series of steps: 1) overnight soak in a 3∶1 chloroform methanol solution to remove lipids; 2) demineralization at room temperature with EDTA (pH 8) for 7–20 days until soft; 3) rinsing 10 times with Milli-Q water and at least one overnight soak; 4) dissolution in Milli-Q water at 110°C; and 5) filtration through muffled fritted glass filters. The filtrate from this process was frozen and lypholized. The purified collagen samples were then submitted as ‘ready to burn’ for δ^13^C and Δ^14^C analyses at the National Ocean Sciences Accelerator Mass Spectrometry Facility (NOSAMS) at the Woods Hole Oceanographic Institution (WHOI).

White sharks are highly migratory [30], [31] with variable feeding habits [32], [33]. As a result, three reference chronologies from the NWA were considered to represent the ocean Δ^14^C rise profile for the region. A coral carbonate chronology from Florida [34] showed a more immediate uptake of bomb radiocarbon compared to the NWA otolith curve [35]. The reference record from validated porbeagle shark data provided a reference for a potential phase lag between ocean radiocarbon curves and vertebral profiles [11]. Radiocarbon values from white shark samples were plotted against the reference chronologies under the assumption of annual band pair deposition.

Where the Δ^14^C rise portion of the vertebral data was displaced to either the right or the left of the reference curves, we shifted the points to bring the entire white shark chronology into alignment. To optimize the alignment, we first fit a linear trend line to the Δ^14^C rise portion of the appropriate reference curve (Florida coral for WS134, and NWA otolith for WS81 and WS105) (Figure S1). Using the resulting equation, we entered each Δ^14^C data point from the vertebral Δ^14^C rise section and located the year corresponding to that point on the reference chronology (Table S1). We found the optimal alignment for each Δ^14^C data point by summing the squared differences between the reference Δ^14^C value for each year and the measured vertebral Δ^14^C from that same year. This calculation was repeated after moving the vertebral years step-wise one year closer each time to the reference years. We identified the optimal shift for each white shark chronology by minimizing the summed squared differences for all the points (Table S2). See supporting information for data and calculations.

Finally, δ^13^C values were also assayed in the collagen samples during the radiocarbon analysis. While δ^13^C values in the ocean did not increase along with radiocarbon values, carbon isoscapes do vary as a function of latitude and distance from the coast and can be a useful tracer of large scale movement patterns [28], [36]. We therefore plotted δ^13^C values by radiocarbon adjusted age and radiocarbon value.

## Results

**Figure 1:**
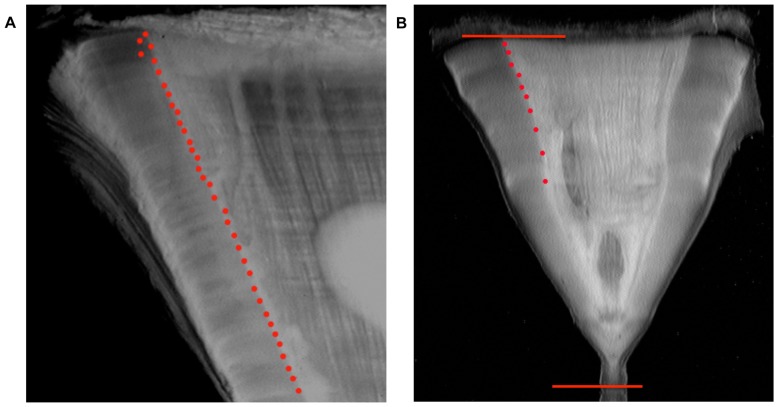
Photomicrographs of sectioned vertebrae. A) Upper section of vertebra taken from WS105. B) WS 100 vertebra; first dot is the birth band. Visible band pairs are marked by dots on the corpus calcareum. The lines indicate the vertebral radius (16.6 mm). Vertebral radius is measured at the angle of the vertebra where the intermedialia meets the corpus calcareum.

**Figure 2:**
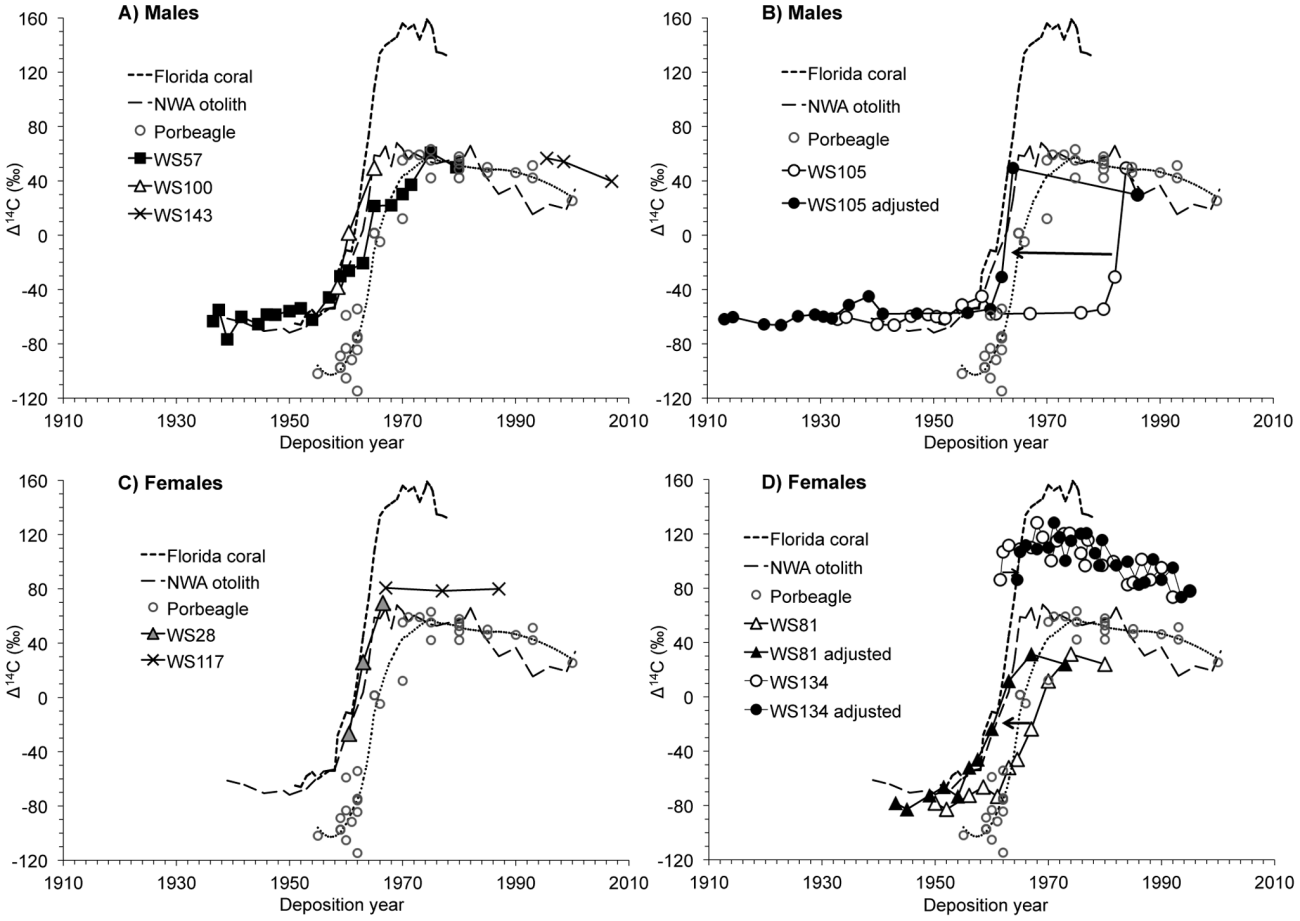
White shark Δ^14^C results compared to three Δ^14^C reference chronologies [11], [31], [32]. Results from male (A, B) and female (C, D) white shark vertebrae. Dotted line is porbeagle data smoothed with a Loess curve. For panels B and D, the arrows indicates the vertebral Δ^14^C curves that had to be shifted to line up with the reference chronologies (white open symbols are initial data, black symbols are data shifted to align with the references).

Band pair counts in vertebral thin sections provided age estimates of 6–35 years for female white sharks and 9–52 years for male white sharks (Figure 1, Table 1). Radiocarbon values in vertebral samples from before the bomb Δ^14^C rise were generally consistent with regional Δ^14^C reference chronologies (mean = −62.5±8.44‰ (SD)) (Figure 2A, B, D). Post-peak radiocarbon values ranged from below the NWA otolith curve to nearly the same amplitude as the coral reference curve from Florida. Female white sharks displayed a broader range in both the absolute magnitude of the Δ^14^C rise and in Δ^14^C post-peak trajectories compared to males (Figure 2A, B, C, D).

**Table 1:** Collection and sampling information for individual sharks.^a^

Shark	Year collected	FL (cm)^b^	Sex	Maturity^c^	Band pairs	Estimated BombΔ^14^C Age	Estimated yearssampled^d^
**WS57**	1981	442	M	M	44	44	1936.5–1979.5
**WS100**	1968	223.5	M	N/A	9	9	1958.5–1965
**WS105**	1986	493	M	M	52	73	1913–1986
**WS143**	2010	222.2	M	I	14	14	1995.5–2007
**WS28**	1967	220.9	F	N/A	6	6	1960.5–1966.5
**WS81**	1983	526	F	N/A	33	40	1943–1973
**WS117**	1988	330	F	N/A	21	21	1967–1987
**WS134**	1996	495.3	F	N/A	35	32	1964.5–1995

^a^ Discrepancies between band pair counts and bomb Δ^14^C age indicate instances where a shift was necessary to align sample Δ^14^C values to reference curves.

^b^ FL, fork length.

^c^ M: mature, I: immature, N/A: information not available.

^d^ Estimates based on band pair counts, and ages estimated from Δ^14^C values when shark trajectories required adjusting.

We found good agreement between the reference curves and band pair counts in three sharks (WS57, WS100, WS28) with nominal ages of 44, 9, and 6 years, respectively. The two youngest white sharks (WS100 and WS28) aligned closely with the coral curve. An older shark (WS57) also aligned with the coral curve up to almost 30 years of age, with a good representation of pre- Δ^14^C rise levels, after which it matched well with the porbeagle reference curve, indicating that vertebral band pairs were indeed deposited on an annual basis (Figure 2A, C). Two other individuals (WS143, WS117) recorded radiocarbon values solely from the enriched post-Δ^14^C rise period with values bracketed by the Δ^14^C reference chronologies (Figure 2A, C). The original Δ^14^C time series of one female (WS134) was plotted with a birth year three years prior to the most rapid increase in Δ^14^C documented, indicating a slight over-estimation of age by the band pair counting, based on the assumption that the coral record provided the best age calibration for this individual (Figure 2D). This assumption was likely appropriate given the similarity of the vertebral Δ^14^C values to the coral chronology, both of which were considerably higher than the other two reference chronologies. In the remaining two sharks - the largest female and male that we examined (WS81, WS105) – the original age estimates led to an offset Δ^14^C chronology for each of these individuals relative to the reference Δ^14^C data. Based on the limits provided by the NWA otolith Δ^14^C reference chronology, each was adjusted (by 7 and 21 years respectively) to a greater age than could be accounted for with the band pair counting. (Figure 2B, D). We kept the terminal data point of WS105 at the year of collection (1986) because it reflected the most recently deposited material in the vertebra. Moving the vertebral values back to the reference curves led to an increase of estimated age to 40 and 73 years for the female and male, respectively (Figure 2B, D).

**Figure 3:**
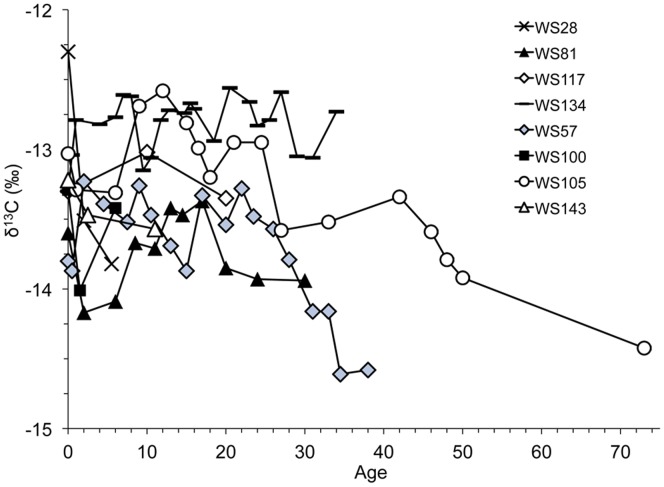
δ^13^C values for individual sharks. Plotted by A) deposition year and B) age as corrected to fit the Δ^14^C reference curves.

**Figure 4:**
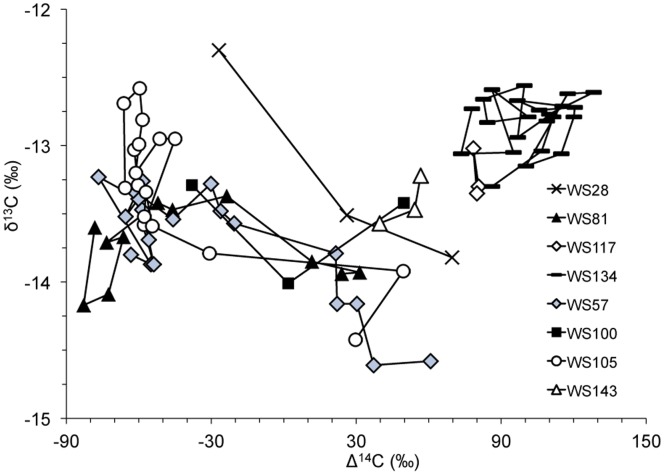
Bivariate plot of δ^13^C versus Δ^14^C for individual sharks.

Examining δ^13^C values as a function of estimated age, all pre-birth δ^13^C values, except for WS28, were clustered with a difference of ∼0.8‰. However, post-birth, δ^13^C values diverged with a tendency towards decline with age but no obvious trend (R = 0.33) (Figure 3). Interestingly, δ^13^C values were positively correlated with Δ^14^C values after (R = 0.80) but not before (R = 0.42) the bomb radiocarbon rise (Figure 4). The maximum difference in δ^13^C values across a single vertebra ranged from 0.33 to 1.84‰ (Table S3). Individual white shark Δ^14^C and δ^13^C (‰) sample values and deposition years are available online (Table S3).

## Discussion

Δ^14^C profiles in vertebrae provided compelling evidence that white sharks are likely to live up to approximately 70 years in the NWA and may live longer. These data stand in contrast to earlier studies in the Pacific and Indian Oceans which suggested that the individual white sharks examined were no older than 23 years [18], [20], [22], [23] with concomitantly faster growth rates. Therefore, either white sharks are living significantly longer and growing slower in the NWA than either the Pacific or Indian Oceans or longevity has been underestimated in previous studies.

The only other study to attempt age validation in white shark vertebrae using Δ^14^C was conducted on samples from the northeastern Pacific Ocean [19]. Results from this earlier study were generally inconclusive for several reasons. The authors used known-age (1 year-old) samples to construct a species-specific Δ^14^C reference that they suggested showed a significant time lag with an otolith reference time series from the Pacific Ocean. However, this interpretation is questionable because no samples were analyzed from the time period between 1959 and 1977 when the Δ^14^C rise occured in the otolith reference chronologies. Kerr et al. [19] did document a phase lag between otolith reference chronologies and samples from assays at the edge of the vertebrae that were assigned chronologically to the year of capture. The phase lag between year-of-capture samples and the Δ^14^C reference chronologies led the authors to suggest that white sharks were consuming some prey with depleted Δ^14^C values from deeper waters in the Pacific. However, the concept of missing growth in the outer margin was not considered because the idea had not yet been described [13].

Our results were substantively different from those derived from the Pacific samples. The NWA white sharks in our study with nominal ages up to 44 years aligned well with reference chronologies, confirming that these sharks deposited one band pair per year up to this age. Moreover, the prey base for NWA white sharks reflected Δ^14^C values commonly found in coastal and epipelagic zones [36]. This observation is consistent with results from the eastern Pacific that found juvenile white sharks tend to stay in shallow water close to the coast [31]. Similar results have also been recently reported for great hammerhead [15], young tiger [14], and young sandbar [16] sharks in the NWA, indicating that these sharks are also using shallow, well-mixed habitats in coastal or oceanic waters.

We found a significant phase lag in larger white sharks that, based on the alignment of young sharks with reference chronologies, was evidence for significant underestimation of age based on band pair counts in these individuals. The Δ^14^C chronologies from these individuals showed that the vertebrae are effectively missing time, on the order of one to two decades. This result is not necessarily surprising as band pair counts appear to also underestimate age in older individuals in other shark species [13], [16], [17], [37]. Given that band pairs are apparently laid down on an annual basis in small to medium sized NWA white sharks, we suggest that the largest individuals may experience a change in the rate of deposition of vertebral material at some point after maturity, or that the band pairs becomes so thin as to be unreadable. This second hypothesis was supported by the shaved terminal sample that we were able to extract from WS105 that was clearly post-bomb and close to the NWA reference value for the year of capture. Andrews et al. [16] and Natanson et al. [17] came to a similar conclusion for sandbar and dusky sharks respectively, in the NWA. Kerr et al. [19] found a similar phase lag in older northeastern Pacific white shark vertebrae that they argued could not be explained by age under-estimation (though variable vertebral growth was mentioned as a possible factor), but rather by assimilation of Δ^14^C-depleted carbon from dietary sources. An alternative interpretation of these data based on the fact that their results showed a phase shift as opposed to a lack of a Δ^14^C rise in the vertebrae suggests that ages may have been underestimated in these individuals. If the sharks were feeding on a significant amount of Δ^14^C-depleted prey, we would expect a noticeably slowed and dampened response to the rise of Δ^14^C in the Pacific, which did not appear to be the case (Figure 1 in [19]). We would also note that it is difficult to constrain the deposition date of material at the terminal edge of large white shark vertebrae even with the fine-scale sampling that we used here. Based on the available data, we cannot determine if the results of our study are applicable to white sharks in other locations as age and growth can vary between different shark populations [11], [13], but further studies are clearly warranted.

While fish otoliths obtain most of their carbon through uptake from DIC [10], [38] diet is likely the primary source of carbon in vertebral collagen of elasmobranchs [11], [37]. This difference in carbon source may lead to problems when comparing radiocarbon curves between inorganic carbonate structures and organic cartilaginous tissues. Equilibration of carbon isotopes incorporated through trophic transfer is likely to be slower than uptake from DIC and this would, in turn, act to reduce the rate of increase and perhaps the amplitude of the radiocarbon rise depending on the variability of tissue turnover rates in food sources. This effect is likely to increase with the trophic level or age of the prey [11], at least to the degree that either variable correlates with carbon turnover rates in muscle tissue of individual prey species. However, NWA white shark records did not show any obvious reduction in the slope of the radiocarbon rise compared to carbonate reference chronologies (though attenuation may be possible and unresolvable in the post-Δ^14^C rise period). The synchronization between the coral reference chronology and WS28 and WS100 demonstrates that white sharks must quickly reach carbon isotopic equilibrium with their diet, or feed on prey that is isotopically equilibrated with ambient DIC. Vertebral samples from other shark species that lag carbonate reference chronologies [11] presumably reach isotopic equilibrium with their environment considerably more slowly than white sharks. Nonetheless, this observation further supports our contention that age under-estimation is the most likely cause of the phase lag between the reference chronologies and the vertebral profiles.

Atlantic white sharks are poorly studied in terms of diet and movement when compared to their Pacific, Australian, and South African counterparts. Post-Δ^14^C rise periods of the white shark profiles revealed some interesting differences among individuals and potentially between sexes that may be related to movement or diet shifts. The post-Δ^14^C rise signal of a location depends on oceanic conditions affecting the diffusion of atmospheric radiocarbon into the sea surface coupled with mixing rates and radiocarbon depth gradients and, therefore, varies significantly both within and across ocean basins [36]. These oceanic conditions as well as biotic factors also impact the δ^13^C signal of a location [36]. We found that male sharks aligned more closely with the NWA otolith reference record than the Florida coral record, suggesting that these individuals spent a significant amount of time in northern shelf waters. However, a similar pattern would also be observed if the sharks shifted to a diet of animals with Δ^14^C–depleted values. Post-Δ^14^C rise radiocarbon values for two females (WS117 and WS 134) sat anywhere from 10–70‰ above the NWA otolith curve, suggesting residency in more southerly and tropical waters than the males in our study.

Our interpretation of habitat differences between sexes and among individuals in this study is reinforced by the post-Δ^14^C rise correlation between δ^13^C and Δ^14^C in the white shark vertebrae; less depleted δ^13^C values are indicative of lower latitudes in the Atlantic and more depleted δ^13^C values are indicative of more northern waters (Fig. 4) [28]. Sex-specific differences in habitat use have been documented for Pacific white sharks [39], as has individual diversity in feeding strategy. Using stable C and N isotopes in vertebrae, Kim et al. [33] found a surprising degree of within and among individual variation attributable to a combination of both differences in diet and movement. Our δ^13^C data also hint at this, with general agreement in early growth, followed by individual differences (Fig. 3). Kerr et al. [19] noted a trend of lower δ^13^C values with increasing age and attributed this to differences in juvenile and adult habitat. While we did not consistently find this pattern, the two oldest individuals, both males (WS57, WS105), exhibited lower δ^13^C values as they aged. Changes in diet also affect δ^13^C values; an increase in trophic level generally corresponds to an enrichment of approximately 1‰ [40]. Based on bulk δ^15^N, which tends to be more sensitive to diet change than δ^13^C, Estrada et al. [41] found apparent size-based trophic shifts in NWA white sharks. It’s likely that the variation in both Δ^14^C and δ^13^C in our study results from a combination of diet and movement differences; more work on the ecology of NWA white sharks is needed to understand and explain the observed variability.

White sharks in our study also displayed marked sexual dimorphism in size at age, assuming our age interpretations are correct. The largest male and female (WS105 and WS81) in this study were similar in size (FLs: 493 cm and 526 cm respectively), yet their ages, as estimated by radiocarbon analyses, differed by up to thirty-three years. WS81, the largest female, is almost a meter longer and yet still four years younger than the second largest male in our study (WS57). The smallest sharks in our study (males: WS100, WS143; female: WS28) are also very similar in size, yet the two males are 3 and 8 years older than the female, respectively. Sexual dimorphism in growth rates is common in lamnids [13], although it is usually thought that larger females are also older. While our sample is limited, the NWA white sharks in this study appear to show the opposite trend. Since the lifetimes and sampling dates of these sharks span several decades, changes in habitat quality may also have influenced this trend.

Assuming a lifespan estimate of 70 years or more, white sharks may be among the longest-lived chondrichthyan fishes [42]. Population projections for white sharks based on earlier age and growth data will, therefore, need to be revisited in the NWA. Modeling of elasmobranch populations has found that age at maturity accounts for most of the variance in population growth rates; sharks that mature late, have long lifespans, and small litters have the lowest population growth rates and longest generation times [43], [44]. While increased overall longevity implies that each individual has greater potential lifetime productivity, modeling studies suggest that the ability of a shark species to recover from fishing pressure is little affected by overall longevity [43], and changes in juvenile survival actually have the greatest effect on population growth rates [44]. We predict that age at maturity for NWA white sharks will be substantially higher than estimates from other areas, using our age data. Earlier work concluded that white sharks have low rebound potential when exposed to fishing pressure [43] and high intrinsic vulnerability to extinction [45]. Thus an increase in age at maturity would make white sharks even more sensitive to fishing pressure than previously thought. While already protected in many nations, even low levels of bycatch mortality are likely to have significant impacts [46] on attempts to rebuild white shark populations from historical over-fishing in the NWA ([47] but see [48]) and potentially other populations in the Pacific and Indian Oceans.

## Supporting Information

Figure S1 **Linear trends fit to the Δ^14^C rise portion of the reference chronologies.** A) Florida coral reference chronology, used to correct WS134. B) NWA otolith reference chronology, used to correct WS81 and WS105.
(DOCX)

Table S1 **Phase lagged Δ^14^C shark values.**
(DOCX)

Table S2 **Calculating the summed squared differences for optimal chronology shifting.** The top line for each chronology is its original placement based on band pair counts. Subsequent lines move the Δ^14^C values step-wise one year closer to their corresponding date on the reference chronology (the values in column 3 from Table S1). Columns “Diffs. 1–4″ refer to the squared differences between the date on the reference chronology and the step-wise adjusted year. “Sum” is the summed squared differences for all points from one shark, and the optimum shift is when this value is minimized; these values have been bolded and starred (*) for each chronology.
(DOCX)

Table S3 **Δ^14^C and δ^13^C (‰) shark sample data listed by individual.**
(DOCX)
