# Supplementary material for: Vertebral Bomb Radiocarbon Suggests Extreme Longevity in White Sharks
Source: PLoS One. 2014 Jan 8;9(1):e84006. doi: 10.1371/journal.pone.0084006 (PMC3885533; doi:10.1371/journal.pone.0084006)

**Figure S1. Linear trends fit to the ∆^14^C rise portion of the reference chronologies.** A) Florida coral reference chronology, used to correct WS134. B) NWA otolith reference chronology, used to correct WS81 and WS105.


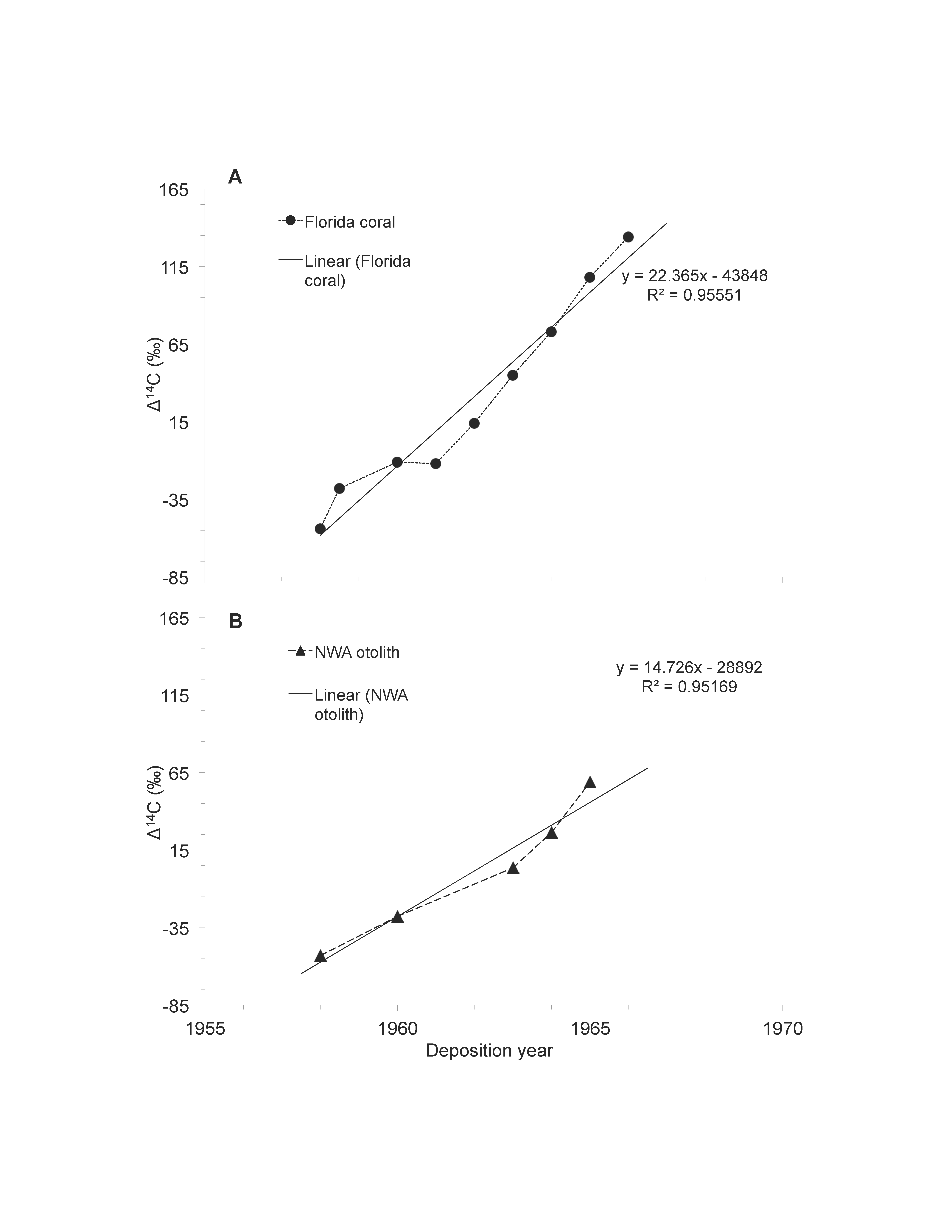

Supplement: Figure S1 — Linear trends fit to the Δ14C rise portion of the reference chronologies. A) Florida coral reference chronology, used to correct WS134. B) NWA otolith reference chronology, used to correct WS81 and WS105. (DOCX) [file pone.0084006.s001.docx]
