# Supplementary material for: Vertebral Bomb Radiocarbon Suggests Extreme Longevity in White Sharks
Source: PLoS One. 2014 Jan 8;9(1):e84006. doi: 10.1371/journal.pone.0084006 (PMC3885533; doi:10.1371/journal.pone.0084006)
Supplement: Table S1 — Phase lagged Δ14C shark values. (DOCX) [file pone.0084006.s002.docx]

**Table S1: Phase lagged ∆^14^C shark values.**

| Shark | Shark ∆^14^C (‰)^[[1]](#footnote-1)^ | Year^[[2]](#footnote-2)^ |
| --- | --- | --- |
| WS81 | -52.09 | 1957.88 |
|  | -46.07 | 1958.29 |
|  | -23.61 | 1959.82 |
|  | 11.65 | 1962.21 |
| WS105 | -54.44 | 1957.72 |
|  | -30.76 | 1959.33 |
|  | 49.47 | 1964.78 |
| WS134 | 86.19 | 1964.42 |
|  | 106.79 | 1965.34 |

1. ∆^14^C values from shark samples that were initially misaligned with the bomb radiocarbon pulse as recorded in the reference chronologies, based on band pair counts. [↑](#footnote-ref-1)
2. The year on the reference trend line corresponding to the shark ∆^14^C value, as determined using the equations from Figure S1. [↑](#footnote-ref-2)
