# Supplementary material for: Vertebral Bomb Radiocarbon Suggests Extreme Longevity in White Sharks
Source: PLoS One. 2014 Jan 8;9(1):e84006. doi: 10.1371/journal.pone.0084006 (PMC3885533; doi:10.1371/journal.pone.0084006)
Supplement: Table S2 — Calculating the summed squared differences for optimal chronology shifting. The top line for each chronology is its original placement based on band pair counts. Subsequent lines move the Δ14C values step-wise one year closer to their corresponding date on the reference chronology (the values in column 3 from Table S1). Columns “Diffs. 1–4″ refer to the squared differences between the date on the reference chronology and the step-wise adjusted year. “Sum” is the summed squared differences for all points from one shark, and the optimum shift is when this value is minimized; these values have been bolded and starred (*) for each chronology. (DOCX) [file pone.0084006.s003.docx]

**Table S2: Calculating the summed squared differences for optimal chronology shifting.**

| Shark | Point 1 | Point 2 | Point 3 | Point 4 | Diff. 1 | Diff. 2 | | Diff. 3 | Diff. 4 | Sum |
| --- | --- | --- | --- | --- | --- | --- | --- | --- | --- | --- |
| WS81 | 1963 | 1964.5 | 1967 | 1970 | 26.2144 | 38.5641 | | 51.5524 | 60.6841 | 177.015 |
|  | 1962 | 1963.5 | 1966 | 1969 | 16.9744 | 27.1441 | | 38.1924 | 46.1041 | 128.415 |
|  | 1961 | 1962.5 | 1965 | 1968 | 9.7344 | 17.7241 | | 26.8324 | 33.5241 | 87.815 |
|  | 1960 | 1961.5 | 1964 | 1967 | 4.4944 | 10.3041 | | 17.4724 | 22.9441 | 55.215 |
|  | 1959 | 1960.5 | 1963 | 1966 | 1.2544 | 4.8841 | | 10.1124 | 14.3641 | 30.615 |
|  | 1958 | 1959.5 | 1962 | 1965 | 0.0144 | 1.4641 | | 4.7524 | 7.7841 | 14.015 |
|  | 1957 | 1958.5 | 1961 | 1964 | 0.7744 | 0.0441 | | 1.3924 | 3.2041 | 5.415 |
|  | 1956 | 1957.5 | 1960 | 1963 | 3.5344 | 0.6241 | | 0.0324 | 0.6241 | **4.815*** |
|  | 1955 | 1956.5 | 1959 | 1962 | 8.2944 | 3.2041 | | 0.6724 | 0.0441 | 12.215 |
|  | 1954 | 1955.5 | 1958 | 1961 | 15.0544 | 7.7841 | | 3.3124 | 1.4641 | 27.615 |
| WS105 | 1980 | 1982 | 1984 |  | 496.3984 | 513.9289 | | 369.4084 |  | 1379.7357 |
|  | 1979 | 1981 | 1983 |  | 452.8384 | 469.5889 | | 331.9684 |  | 1254.3957 |
|  | 1978 | 1980 | 1982 |  | 411.2784 | 427.2489 | | 296.5284 |  | 1135.0557 |
|  | 1977 | 1979 | 1981 |  | 371.7184 | 386.9089 | | 263.0884 |  | 1021.7157 |
|  | 1976 | 1978 | 1980 |  | 334.1584 | 348.5689 | | 231.6484 |  | 914.3757 |
|  | 1975 | 1977 | 1979 |  | 298.5984 | 312.2289 | | 202.2084 |  | 813.0357 |
|  | 1974 | 1976 | 1978 |  | 265.0384 | 277.8889 | | 174.7684 |  | 717.6957 |
|  | 1973 | 1975 | 1977 |  | 233.4784 | 245.5489 | | 149.3284 |  | 628.3557 |
|  | 1972 | 1974 | 1976 |  | 203.9184 | 215.2089 | | 125.8884 |  | 545.0157 |
|  | 1971 | 1973 | 1975 |  | 176.3584 | 186.8689 | | 104.4484 |  | 467.6757 |
|  | 1970 | 1972 | 1974 |  | 150.7984 | 160.5289 | | 85.0084 |  | 396.3357 |
|  | 1969 | 1971 | 1973 |  | 127.2384 | 136.1889 | | 67.5684 |  | 330.9957 |
|  | 1968 | 1970 | 1972 |  | 105.6784 | 113.8489 | | 52.1284 |  | 271.6557 |
|  | 1967 | 1969 | 1971 |  | 86.1184 | 93.5089 | | 38.6884 |  | 218.3157 |
|  | 1966 | 1968 | 1970 |  | 68.5584 | 75.1689 | | 27.2484 |  | 170.9757 |
|  | 1965 | 1967 | 1969 |  | 52.9984 | 58.8289 | | 17.8084 |  | 129.6357 |
|  | 1964 | 1966 | 1968 |  | 39.4384 | 44.4889 | | 10.3684 |  | 94.2957 |
|  | 1963 | 1965 | 1967 |  | 27.8784 | 32.1489 | | 4.9284 |  | 64.9557 |
|  | 1962 | 1964 | 1966 |  | 18.3184 | 21.8089 | | 1.4884 |  | 41.6157 |
|  | 1961 | 1963 | 1965 |  | 10.7584 | 13.4689 | | 0.0484 |  | 24.2757 |
|  | 1960 | 1962 | 1964 |  | 5.1984 | 7.1289 | | 0.6084 |  | 12.9357 |
|  | 1959 | 1961 | 1963 |  | 1.6384 | 2.7889 | | 3.1684 |  | **7.5957*** |
|  | 1958 | 1960 | 1962 |  | 0.0784 | 0.4489 | | 7.7284 |  | 8.2557 |
|  | 1957 | 1959 | 1961 |  | 0.5184 | 0.1089 | | 14.2884 |  | 14.9157 |
| WS134 | 1961.5 | 1962 |  |  | 8.5264 | 11.1556 | |  |  | 19.682 |
|  | 1962.5 | 1963 |  |  | 3.6864 | 5.4756 | |  |  | 9.162 |
|  | 1963.5 | 1964 |  |  | 0.8464 | 1.7956 | |  |  | 2.642 |
|  | 1964.5 | 1965 |  |  | 0.0064 | 0.1156 | |  |  | **0.122*** |
|  | 1965.5 | 1966 |  |  | 1.1664 | 0.4356 | |  |  | 1.602 |
|  | 1966.5 | 1967 |  |  | 4.3264 | 2.7556 |  | |  | 7.082 |

The top line for each chronology is its original placement based on band pair counts. Subsequent lines move the ∆^14^C values step-wise one year closer to their corresponding date on the reference chronology (the values in column 3 from Table S1). Columns “Diffs. 1-4” refer to the squared differences between the date on the reference chronology and the step-wise adjusted year. “Sum” is the summed squared differences for all points from one shark, and the optimum shift is when this value is minimized; these values have been bolded and starred (*) for each chronology.
