# Supplementary material for: Vertebral Bomb Radiocarbon Suggests Extreme Longevity in White Sharks
Source: PLoS One. 2014 Jan 8;9(1):e84006. doi: 10.1371/journal.pone.0084006 (PMC3885533; doi:10.1371/journal.pone.0084006)
Supplement: Table S3 — Δ14C and δ13C (‰) shark sample data listed by individual. (DOCX) [file pone.0084006.s004.docx]

**Table S3: ∆^14^C and δ^13^C (‰) shark sample data listed by individual.**

| Shark | Year^[[1]](#endnote-1)^ (band pair) | Year (∆^14^C) | Age of formation^[[2]](#endnote-2)^ | ∆^14^C (‰) | δ^13^C (‰)^[[3]](#endnote-3)^ | Overall difference in δ^13^C (‰)^[[4]](#endnote-4)^ |
| --- | --- | --- | --- | --- | --- | --- |
| WS57 |  | 1936.5 | 0 | -63.27 | -12.62 | 1.49 |
|  |  | 1937.5 | 1 | -54.97 | -13.3 |  |
|  |  | 1939 | 2.5 | -76.67 | -13.37 |  |
|  |  | 1941.5 | 4.5 | -60.13 | -12.73 |  |
|  |  | 1944.5 | 7.5 | -65.48 | -12.89 |  |
|  |  | 1946 | 9 | -58.44 | -13.02 |  |
|  |  | 1947.5 | 10.5 | -58.62 | -12.76 |  |
|  |  | 1950 | 13 | -55.90 | -12.97 |  |
|  |  | 1952 | 15 | -53.83 | -13.19 |  |
|  |  | 1954 | 17 | -62.25 | -13.37 |  |
|  |  | 1957 | 20 | -45.81 | -12.83 |  |
|  |  | 1959 | 22 | -30.16 | -13.04 |  |
|  |  | 1960.5 | 23.5 | -26.14 | -12.78 |  |
|  |  | 1963 | 26 | -20.54 | -12.98 |  |
|  |  | 1965 | 28 | 21.54 | -13.07 |  |
|  |  | 1968 | 31 | 22.07 | -13.29 |  |
|  |  | 1970 | 33 | 30.30 | -13.66 |  |
|  |  | 1971.5 | 34.5 | 37.20 | -13.66 |  |
|  |  | 1975 | 38 | 60.79 | -14.11 |  |
|  |  | 1979.5 | 42.5 | 50.05 | -14.08 |  |
| WS100 |  | 1958.5 | 0 | -37.99 | -12.79 | 0.72 |
|  |  | 1960.5 | 1.5 | 1.83 | -13.51 |  |
|  |  | 1965 | 6 | 49.69 | -12.92 |  |
| WS105 | 1933 | 1913 | 0 | -61.87 | -12.53 | 1.843 |
|  | 1934.5 | 1914.5 | 1 | -60.34 | -12.79 |  |
|  | 1940 | 1920 | 6 | -65.67 | -12.81 |  |
|  | 1943 | 1923 | 9 | -66.21 | -12.19 |  |
|  | 1946 | 1926 | 12 | -59.65 | -12.08 |  |
|  | 1949 | 1929 | 15 | -58.49 | -12.31 |  |
|  | 1950.5 | 1930.5 | 16.5 | -59.96 | -12.49 |  |
|  | 1952 | 1932 | 18 | -61.23 | -12.70 |  |
|  | 1955 | 1935 | 21 | -51.37 | -12.45 |  |
|  | 1958.5 | 1938.5 | 24.5 | -44.98 | -12.45 |  |
|  | 1961 | 1941 | 27 | -57.85 | -13.08 |  |
|  | 1967 | 1947 | 33 | -57.74 | -13.02 |  |
|  | 1976 | 1956 | 42 | -57.07 | -12.84 |  |
|  | 1980 | 1960 | 46 | -54.44 | -13.09 |  |
|  | 1982 | 1962 | 48 | -30.76 | -13.29 |  |
|  | 1984 | 1964 | 50 | 49.47 | -13.42 |  |
|  | 1986 | 1986 | 73 | 29.71 | -13.923 |  |
| WS143 |  | 1995.5 | 0 | 56.67 | -12.72 | 0.35 |
|  |  | 1998.5 | 2.5 | 54.20 | -12.97 |  |
|  |  | 2007 | 11 | 39.71 | -13.07 |  |
| WS28 |  | 1960.5 | 0 | -26.84 | -11.80 | 1.52 |
|  |  | 1963 | 2 | 26.09 | -13.01 |  |
|  |  | 1966.5 | 5.5 | 69.66 | -13.32 |  |
| WS81 | 1950 | 1943 | 0 | -78.20 | -13.10 | 0.8 |
|  | 1952 | 1945 | 2 | -82.82 | -13.67 |  |
|  | 1956 | 1949 | 6 | -72.57 | -13.59 |  |
|  | 1958.5 | 1951.5 | 8.5 | -66.36 | -13.17 |  |
|  | 1961 | 1954 | 11 | -73.33 | -13.21 |  |
|  | 1963 | 1956 | 13 | -52.09 | -12.92 |  |
|  | 1964.5 | 1957.5 | 14.5 | -46.07 | -12.97 |  |
|  | 1967 | 1960 | 17 | -23.61 | -12.87 |  |
|  | 1970 | 1963 | 20 | 11.65 | -13.35 |  |
|  | 1974 | 1967 | 24 | 31.40 | -13.43 |  |
|  | 1980 | 1973 | 30 | 23.98 | -13.44 |  |
| WS117 |  | 1967 | 0 | 80.68 | -12.80 | 0.33 |
|  |  | 1977 | 10 | 78.47 | -12.52 |  |
|  |  | 1987 | 20 | 79.96 | -12.85 |  |
| WS134 | 1961.5 | 1964.5 | 0 | 86.19 | -12.8 | 0.71 |
|  | 1962 | 1965 | 1 | 106.79 | -12.54 |  |
|  | 1963 | 1966 | 2 | 111.55 | -12.29 |  |
|  | 1965 | 1968 | 4 | 108.89 | -12.32 |  |
|  | 1967 | 1970 | 6 | 109.92 | -12.27 |  |
|  | 1968 | 1971 | 7 | 128.24 | -12.11 |  |
|  | 1969 | 1972 | 8 | 117.53 | -12.12 |  |
|  | 1970.5 | 1973 | 9 | 100.27 | -12.65 |  |
|  | 1971.5 | 1974 | 10 | 114.90 | -12.56 |  |
|  | 1972.75 | 1975.75 | 11.75 | 120.11 | -12.29 |  |
|  | 1973.75 | 1976.75 | 12.75 | 120.48 | -12.22 |  |
|  | 1975.75 | 1978.25 | 14.25 | 105.75 | -12.24 |  |
|  | 1976.5 | 1979 | 15 | 96.68 | -12.17 |  |
|  | 1977 | 1979.5 | 15.5 | 115.45 | -12.21 |  |
|  | 1979.5 | 1982 | 18 | 96.88 | -12.44 |  |
|  | 1981.5 | 1984 | 20 | 99.60 | -12.06 |  |
|  | 1984 | 1986 | 22 | 82.64 | -12.16 |  |
|  | 1985 | 1987 | 23 | 84.30 | -12.33 |  |
|  | 1986.5 | 1988.5 | 24.5 | 101.23 | -12.29 |  |
|  | 1988 | 1990 | 26 | 86.20 | -12.09 |  |
|  | 1990 | 1992 | 28 | 95.09 | -12.55 |  |
|  | 1992 | 1993.5 | 29.5 | 73.43 | -12.56 |  |
|  | 1995 | 1995 | 31 | 77.92 | -12.23 |  |

1. Year deposited according to band pair count only listed for samples requiring a shift in order to align sample ∆^14^C values to reference curves. [↑](#endnote-ref-1)
2. Estimates based on band pair counts, and ages estimated from ∆^14^C values when shark trajectories required adjusting. [↑](#endnote-ref-2)
3. The δ^13^C values of the samples (mean = -12.82 ± 0.055‰ (SE)) are typical of metabolically derived carbon [11, 35]. [↑](#endnote-ref-3)
4. The overall difference in δ^13^C is the maximum difference between δ^13^C measurements on an individual vertebra. [↑](#endnote-ref-4)
